# A Plausible Fallacy

**Published:** 1895-09

**Authors:** Edmund Carleton

**Affiliations:** New York, N. Y.


### A PLAUSIBLE FALLACY.

EDMUND CARLETON, M. D., NEW YORK, N. Y.

A number of years ago it began to be said among us that a high potency of any substance would antidote the effects upon the human system, of the same substance in a crude form; and more of the same sort. If any patient had gonorrhœa or syphilis let him swallow a high potency of the pus taken from another individual having gonorrhœa or syphilis, and presto! he would be well.

All this excited but a languid interest at first, and was regarded as the talk of a very few active and diffuse individuals. But the noise did not stop. Printer's ink was shed without stint. Alleged "provings" and "clinical reports" were foisted upon an unsuspecting audience; and now the credulity of too many Hahnemannians has been affected by all these influences. Let us compare the theory with Hahnemann's teachings.

We find in *The Organon* (4th Am. ed., p. 86, with note): "Frozen sour-kraut is frequently applied to a limb that is recently frozen, or, sometimes, it is rubbed with snow."

It is on such examples of domestic practice that M. Lux founds his so-called mode of cure, by *identicals* and *idem*, which he calls *isopathy*, which some eccentric-minded persons have already adopted as the *ne plus ultra* of a healing art, without knowing how they can carry it out in practice. But, if we examine these instances attentively we find that they do not bear out these views.

The purely physical powers differ in the nature of their action on the living organism from those of a dynamic medical kind.

Heat or cold of the air that surrounds us, or of the water, or of our food and drink occasion (*as heat and cold*) *of themselves* no absolute injury to a healthy body; heat and cold are, in their alternations, essential to the maintenance of healthy life; consequently they are not of themselves medicine. Heat and cold, therefore, act as curative agents in affections of the body, not by virtue of their essential nature (not, therefore, as heat and cold *per se*, not as things hurtful in themselves, as are the drugs, Rhubarb, China, etc., even in the smallest doses), but *only* by virtue of their greater or smaller *quantity*—that is, according to their degree of temperature. * * *

That serpent's bites, * * * are most certainly cured by portions of the serpents must remain a mere fable of a former age, until such an improbable assertion is authenticated by indubitable observation and experience, which it certainly never will be. That, in fine, the saliva of a mad dog given to a patient laboring under hydrophobia (in Russia) *is said* to have cured him. That "*is said*" would not seduce any conscientious physician to imitate such a hazardous experiment. * * *

In section 24, Hahnemann says: "There remains, accordingly, no other method of applying medicines profitably in diseases than the homœopathic." * * * And in section 26 he puts these words in italics: " * * * *a dynamic disease in the living economy of man is extinguished in a permanent manner by another that is more powerful, when the latter (without being of the same species) bears a strong resemblance to it in its mode of manifesting itself.*"

Mark well these italics. They impale the fallacy. "WITHOUT BEING OF THE SAME SPECIES!" Print them in capitals. But those who resort to the method of identicals *do not change the species.* A victim of neuralgia and *Quininism* presented more symptoms of *Sulphate of Quinine* than of anything else, of course. The next nearest proved agent in the scale of similarity was *Arsenicum.* With difficulty the attending physician was persuaded not to give potentized *Chininum-sulphuricum,* but to give *Arsenicum.* Slow improvement followed. It was not rapid enough to suit. *Arsenicum* was abandoned; *Chininum-sulphuricum* was given; the patient died promptly.

Section 32 of *The Organon* says: " * * * Every real medicine will, at *all* times and under *every* circumstance, work upon *every* living individual, and excite in him the symptoms that are peculiar to it. * * * " Always the same symptoms. Consequently all the symptoms of that medicine are included. All are recorded together in the *Materia Medica.* In Allen's *Encyclopedia* there are 1,076 symptoms recorded under *Chininum-sulphuricum,* with four additional pages devoted to conditions and amelioration. Fifty-four authorities are cited. Hering devotes fourteen pages of his *Guiding Symptoms* to this drug. The provings are from many sources and from differing degrees of strength of the substance proved; but they are all *Chininum-sulphuricum.* They are recorded together. Therefore, if an individual presents symptoms of the drug named, and it is known that they have been produced by that drug, the physician who shall give as a remedy the same drug in any strength is practicing isopathy and not Homœopathy.

*Ex uno disce omnes.* We are not to attempt to cure scarlet fever with a potency made from the scales of another scarlet fever patient, which is isopathy; but are to select *Belladonna*, or some other agent which a careful search shows to be *similar* (not *identical*) to the case in hand, which is Homœopathy. And the same is to be said of gonorrhœa, syphilis, *et id omne genus*. Cheap and nasty generalizing will not answer.

Great efforts are being made to find respectable authority for the employment of the same instead of the similar, with especial reference to antidoting drug diseases. This has already been alluded to. It is said that drowning men clutch at straws. Well, the straw that these drowning men clutch at is found in a foot-note of *The Organon*, page 122, as follows: "But, even granting this could be done, which would certainly be a most valuable discovery. * * * " Observe the words, "*even granting*" and "*would be*." Not much *is* about them. No authority at all, but a gentle admonition. The *Chronic Diseases* and the *Materia Medica Pura* afford no succor whatever to the drowning men. On the contrary, the forty-nine remedies given in the *Chronic Diseases* and the sixty-seven in the *Materia Medica Pura* furnish a multitude of instances of exact statement how to antidote the effects of drugs, and in every case some other drug than the one to be antidoted is named—not the same drug in higher potency. The very first remedy named in the *Materia Medica*, *Aconitum*, is, by plain directions, to be antidoted by "vegetable acids and wine," not by *Aconite* in high potency. Those who talk of antidoting *Rhus* with *Rhus* may profitably read and reflect upon these words of Hahnemann: "The injurious effects of an erroneous selection are often removable by *Bryonia*, sometimes by *Sulphur*, at other times by *Camphor* or *raw Coffee*, according to the untoward symptoms produced." No mention of *Rhus* in higher potency. How many medicines are antidoted by *Camphor* and *Nux-vomica!* It is not necessary to put in array more proof from Hahnemann.

Equally unsuccessful is the endeavor to press Lippe into the service. Not a word can be found in his writings to favor the heresy. But he did at times speak very energetically against it, to which the writer of this paper bears personal testimony. On page 324 of his *Materia Medica* occur these words: "The best antidote for too large doses or too intense symptoms (of *Kali-bichromicum*) is its relative, *Pulsatilla*. * * *" And again, on page 338, read the following:

"After abuse of *Mercury*, pain in the bones, catarrh; against the abuse of *Kali-hydriodicum* in massive doses, *Hepar-s-c.* is the best antidote."

Hering has been misquoted in the endeavor to range him on the side of isopathy. He is claimed as favoring the use of *Opium* and *Mercury* high to antidote the same substances crude or too low. The text of *Opium* in *The Guiding Symptoms* offers not the slightest foundation for such a claim. It is puzzling to understand why it ever was brought forward. As for *Mercury*, we find in Vol. VII, page 406, under Relations, this paragraph: "Antidoted by: *Aurum*, *Asaf.*, *Bellad.*, *Carbo-veg.*, *Cinchona*, *Dulcam.*, *Ferrum*, *Guaiac.*, *Hepar*, *Iodum*, *Kali-chlor.*, *Kali-hyd.*, *Laches.*, *Mezer.*, *Nitr-ac.*, *Staphis.*, *Stillingia*, *Sulphur*, and, according to Guernsey, by *Mercur.* itself, high, all symptoms agreeing." This posthumous allusion to the claim of another author, and put in guarded language at that, is all! There is not so much as a straw left.

It is hard work to practice the true healing art. Let those who are tempted to try easy methods remember that they all are rejected by Hahnemann. Only the painstaking, homœopathic method will do.

Adjourned to 8.30 P. M.

